# Quantitative assessment of upper limb motor function in Multiple Sclerosis using an instrumented Action Research Arm Test

**DOI:** 10.1186/1743-0003-11-67

**Published:** 2014-04-18

**Authors:** Ilaria Carpinella, Davide Cattaneo, Maurizio Ferrarin

**Affiliations:** 1 Biomedical Technology Department, Found. Don C. Gnocchi Onlus, IRCCS, Via Capecelatro 66, 20148 Milan, Italy; 2 LaRiCE: Gait and Balance Disorders Laboratory, Department of Neurorehabilitation, Found. Don C. Gnocchi Onlus, IRCCS, Via Capecelatro 66, 20148 Milan, Italy

**Keywords:** Multiple Sclerosis, Upper limb function, ARAT, Inertial sensors

## Abstract

**Background:**

Arm impairment in Multiple Sclerosis (MS) is commonly assessed with clinical scales, such as Action Research Arm Test (ARAT) which evaluates the ability to handle and transport smaller and larger objects. ARAT provides a complete upper limb assessment, as it considers both proximal arm and hand, but suffers from subjectivity and poor sensitivity to mild impairment. In this study an instrumented ARAT is proposed to overcome these limitations and supplement the assessment of arm function in MS.

**Methods:**

ARAT was executed by 12 healthy volunteers and 21 MS subjects wearing a single inertial sensor on the wrist. Accelerometers and gyroscopes signals were used to calculate the duration of each task and its sub-phases (reaching, manipulation, transport, release and return). A jerk index was computed to quantify movement smoothness. For each parameter, z-scores were calculated to analyze the deviation from normative data. MS subjects were clinically assessed with ARAT score, Nine-Hole Peg test (9HPT) and Fahn Tremor Rating Scale (FTRS).

**Results:**

ARAT tasks executed by MS patients were significantly slower (duration increase: 70%) and less smooth (jerk increase: 16%) with respect to controls. These anomalies were mainly related to manipulation, transport and release sub-movements, with the former showing the greatest alterations. A statistically significant decrease in movement velocity and smoothness was also noticed in patients with normal ARAT score. Z-scores related to duration and jerk were strongly correlated with ARAT rating (r < -0.80, p < 0.001) and 9HPT (r < -0.75, p < 0.001) and were significantly different among MS sub-groups with different levels of arm impairments (p < 0.001). Moreover, Z-score related to manipulation-phase jerk was significantly correlated with the FTRS rating of intention tremor (r = 0.84, p < 0.001).

**Conclusions:**

The present study showed that the proposed method is able to discriminate between control and MS groups and to reveal subtle arm alterations not detectable from ARAT score. Validity was shown by high correlations between instrumental variables and clinical ratings. These results suggested that instrumented ARAT could be a valid quick and easy-to-use method for a sensitive quantification of arm function in MS. Inclusion of finger-mounted sensors could complement present findings and provide further indications about hand function in MS.

## Background

Nearly 75% of people with Multiple Sclerosis (MS) experience upper limb dysfunctions mainly related to tremor, coordination deficit and muscle weakness [1]. These symptoms have been shown to highly reduce the quality of life [2,3] and some recent studies suggested that neuromotor rehabilitation may be useful to reduce these alterations by improving manual dexterity, arm strength and execution of the activities of daily living (ADL) [4-8]. For this reason, it is essential to have quantitative and sensitive tools to evaluate upper limb function in MS and to monitor the possible effects of the applied treatment.

In clinical settings, upper extremity function is generally assessed through standardized ordinal scales or timed tests. Some of the standard assessment tools used for MS patients are Scripps Neurological Rating Scale [9], Fugl-Meyer Assessment [10,11], Action Research Arm Test [11,12], TEMPA [13], Box & Block Test [11,14], Nine Hole Peg Test [14,15] and Jebsen-Taylor Test [13,16]. From a statistical point of view ordinal scales are reliable [11] and sensitive for measuring gross changes in motor performance but have less sensitivity to smaller and more specific changes [17,18]. They also suffer from poor sensitivity to mild impairment because of a significant ceiling effect [10,19]. Furthermore, despite the extensive experience in using ordinal scales by clinicians, their rating is subjective. Timed tests are more objective than ordinal scales, as the final score is represented by the time to complete the task. They are also less influenced by ceiling effect but they do not provide a complete assessment of upper limb function, as they involve only partial movements of proximal arm, mainly focusing on hand movements (e.g. Jebsen-Taylor Test) and gross/fine manual dexterity (Box & Block and Nine Hole Peg Test). Moreover, all the above tests evaluate each task as a whole and do not provide more specific and detailed indications of the sub-phases which compose the movement (e.g. manipulation of an object, transport and release). For all these reasons, there is a need to develop new easy-to-use measurement tools which can provide more objective and detailed evaluation of upper limb function, necessary for the analysis of the specific deficit of each subject and for the definition of personalized rehabilitation treatments.

In the last years, the use of wearable inertial measurement units (IMU) for assessment of motor function has grown significantly, since they are relatively cheap and, in contrast with optoelectronic or video based systems, easily allow for measurements outside the motion laboratory [20]. Recently, IMU have been used for the instrumentation of several clinical tests for balance and gait, such as Timed Up and Go Test [21,22] and posturography [23]. As for upper limb, preliminary results are available for stroke subjects evaluated with instrumented Fugl-Meyer Assessment [24] and Wolf Motor Function Test [25,26] and for parkinsonian patients executing three items of UPDRS scale with a finger mounted inertial sensor [27]. To our knowledge, no studies exist about the instrumentation of a clinical test for the evaluation of upper limb in subjects with MS.

Aim of the present study was the development and the application on a group of healthy subjects and on persons with MS of a quantitative method for upper limb assessment based on a single inertial sensor which records linear accelerations and angular velocities during the execution of the Action Reasearch Arm Test (ARAT). This clinical test was chosen among the others because it has been frequently used to rate subjects with stroke [11], spinal cord injuries [28] and multiple sclerosis [5,7,8,11], it requires relatively short administration time (8–10 minutes [29]) and it allows the evaluation of both arm and hand during the execution of functional tasks very similar to the ADL. Moreover, the well defined set up [30] makes possible the careful description and standardization of the tasks. In particular, the present work had three main purposes: i) evaluation of the method’s ability to discriminate motor performances of MS subjects from that of healthy controls, ii) evaluation of the method’s ability to detect subtle alterations not visible from clinical scales and iii) analysis of the validity of proposed indexes for evaluating upper limb impairment.

## Methods

### Subjects

A consecutive sample of twenty-one subjects with MS (9 women and 12 men, mean age: 47.4 ± 9.0 years) and twelve healthy volunteers with comparable age (5 women and 7 men, mean age: 44.3 ± 9.5 years) participated in the study. Demographic and clinical characteristics of MS group are shown in Table 1. MS patients fulfilled the following inclusion criteria: a definite diagnosis of MS according to McDonald criteria [31], Expanded Disability Status Scale [32] <9, Mini-Mental State Examination [33] >24. Exclusion criterion for the control group was the presence of neurological, rheumatic or orthopedic disorders which might interfere with the protocol. All subjects signed an informed consent to the protocol which was conformed to the standards for human experiments set by the Declaration of Helsinki and was approved by the local Ethical Committee.

**Table 1:** Demographic and clinical characteristics of MS subjects

**Subject**	**Age [years]**	**Gender [F/M]**	**MS type [RR/PP/SP]**	**Time since diagnosis [years]**	**EDSS [points]**
S1	69	F	RR	31	6
S2	43	M	RR	15	4.5
S3	57	F	RR	32	6.5
S4	44	M	RR	1	6.5
S5	51	M	SP	29	7.5
S6	45	M	PP	4	6
S7	58	M	SP	14	7
S8	25	F	RR	3	2.5
S9	51	F	SP	25	7.5
S10	40	M	RR	1	2
S11	39	F	SP	23	8
S12	38	F	RR	5	6.5
S13	44	F	SP	23	8.5
S14	55	M	PP	33	6
S15	50	M	PP	18	6.5
S16	38	M	RR	5	6
S17	54	F	RR	27	6
S18	46	F	PP	8	7.5
S19	61	M	SP	31	5.5
S20	45	M	SP	9	6.5
S21	43	M	RR	2	6.5
**Median**	**45**	**9 F/12 M**	**10RR/4PP/7SP**	**15**	**7**
**Min-Max**	**25-69**			**1-33**	**2-8.5**

RR: relapsing remitting; PP: primary progressive; SP: secondary progressive; EDSS: Expanded Disability Status Scale (0–10).

### Experimental protocol

Subjects were tested during the execution of ARAT, a clinical test that evaluates proximal and distal upper limb function [12]. MS subjects performed ARAT with their most affected arm (worst performance at Nine Hole Peg test). Following this criterion, 10 patients (48%) executed ARAT with the dominant side, while the remaining 11 subjects (52%) with their non-dominant arm. To maintain a similar proportion in the control group, dominant side was tested in 6 healthy subjects (50%) and non-dominant side in the remaining 6 volunteers (50%).

**Figure 1:**
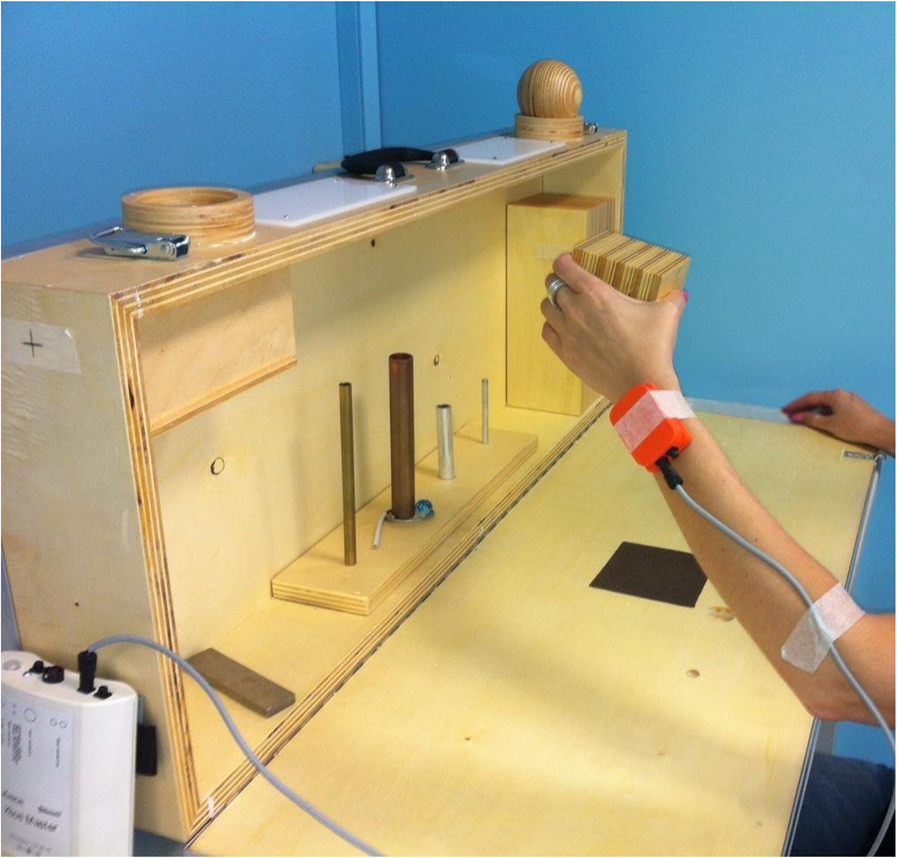
**Experimental set-up.** Example of a subject executing item 4 of ARAT with an inertial measurement unit mounted on the wrist.

ARAT consists of 19 items organized in 4 sections: Grasp, Grip, Pinch and Gross movements (see Table 2). In the present study all ARAT items were performed. Subjects sat upright on a chair with a firm back and no armrests. A table was placed in front of them at a distance of 15 cm from anterior torso and at mid-abdomen height. The objects to be moved were placed on the table in front of the subject, one at a time during the appropriate test. The tested hand was placed pronated, immediately lateral to the object, in correspondence of a starting position marked with tape strips. Upon the verbal go-signal from the physical therapist, subjects executed the specific task and then returned their hand to the starting position. A detailed description of all ARAT tasks is reported in [30]. In summary, Grasp section (items 1–6) required the subject to grasp, transport and then release each object (block, ball, stone) onto the top of a 37-cm-high shelf placed 25 cm away from the proximal edge of the table (see Figure 1). Grip section (items 7–10) required to pour water from one glass to the other (item 7), to horizontally displace 2 different sized alloy tubes from a starting peg to a target peg (items 8–9) and to horizontally displace a washer from a tin to a bolt (item 10). As for Pinch section (items 11–16), the subject was asked to grasp a ball bearing or a marble from a tin lid, transport and than release it into a target tin lid placed on the shelf. Finally, Gross movements section (items 17–19) required the subject to move upper arm and touch the back and the top of the head (item 17–18) and the mouth (item 19). ARAT was administered using standardized commercial equipment (http://www.aratest.eu).

**Table 2:** The action research arm test

**Task Number**	**Item**
	**Grasp Subscale**
1	Block, 10 cm^3^
2	Block, 2.5 cm^3^
3	Block, 5 cm^3^
4	Block, 7.5 cm^3^
5	Cricket ball
6	Sharpening stone
	**Grip subscale**
7	Pour water from one glass to another
8	Displace 2.25-cm alloy tube from one side of table to the other
9	Displace 1-cm alloy tube from one side of table to the other
10	Put washer over bolt
	**Pinch subscale**
11	Ball bearing, held between ring finger and thumb
12	Marble, held between index finger and thumb
13	Ball bearing, held between middle finger and thumb
14	Ball bearing, held between index finger and thumb
15	Marble, held between ring finger and thumb
16	Marble, held between middle finger and thumb
	**Gross movement subscale**
17	Hand to behind the head
18	Hand to the top of the head
19	Hand to the mouth

The performance of each task was scored by the physical therapist on a 4-point ordinal scale, with 0 = unable to complete any part of the task, 1 = the task is only partially completed, 2 = the task is completed but with great difficulty and/or in an abnormally long time, and 3 = the movement is performed normally. The maximum ARAT score is 57 points, which means normal upper limb function.

Before ARAT execution, MS subjects were clinically evaluated with Nine Hole Peg Test (HPT) [14] and Fahn’s Tremor Rating Scale (FTRS) [34]. The 9HPT evaluates hand dexterity and requires the subject to place nine pegs in nine holes and then remove them from the pegboard, as quickly as possible. Subjects are scored on the amount of time they take to place and remove all nine pegs. Alternatively, the score can be expressed as the number of pegs moved per second [35]. The FTRS is a 5-point ordinal scale which rates tremor severity by body part from 0 (no tremor) to 4 (severe tremor). In the present study, FTRS was used for the clinical assessment of upper limb postural and intention tremor. In particular, intention tremor, that is the exacerbation of kinetic tremor towards the end of a goal directed movement [36], was evaluated with finger-to-nose test while postural tremor was assessed by asking subjects to hold their arms outstretched in front of them against gravity for 10 seconds. All clinical tests (ARAT, 9HPT and FTRS) were administered in a single 30-minute session and were rated by one physical therapist with more than 20 years of experience in the field of motor rehabilitation.

### Experimental equipment

Subjects performed all ARAT tasks wearing a single inertial measurement unit (MTX, Xsens, The Netherlands) mounted on the dorsal side of the forearm at wrist level (Figure 1). The IMU consists of a 3D accelerometer (±5 g range), a 3D gyroscope, (±1200°/s range) and a 3D magnetometer (±750 mGauss). The sensor orientation in 3D space, in particular the rotation matrix, was estimated from raw signals by a sensor fusion algorithm based on Kalman Filter implemented on a Digital Signal Processor embedded in the sensor housing [37]. The sensor was connected via cable to a data transmitter (Xbus Master) located on the table. Orientation data and raw signals from accelerometers, gyros and magnetometers were acquired with a sampling frequency of 50 Hz.

Four healthy subjects were tested with the inertial sensor and, simultaneously, with an optoelectronic motion analysis system (Smart, EMotion, Italy), assumed as a gold standard. The system consisted in nine infrared video cameras working at a sampling rate of 200 Hz. The working volume (70 × 70 × 70 cm^3^) was calibrated to provide an accuracy of less than 0.3 mm. Thirteen retro-reflective hemispheric markers with a diameter of 6 mm were used: one marker was attached on the upper surface of the IMU in correspondence of its mid-point, while the other twelve markers were positioned on the objects to be moved (ten markers for items 1 to 10, two markers for items 11 to 16).

### Data processing

After data acquisition, IMU signals were processed to characterize upper limb function in MS subjects and healthy controls. Data processing consisted in: 1) movement segmentation and 2) extraction of quantitative parameters. All procedures were implemented using MATLAB® software (The MathWorks, Inc., Natick, MA).

### Movement segmentation

The first step consisted in the segmentation of each ARAT task into basic sub-movements. For items 1 to 16 (Grasp, Grip and Pinch sections), movements were subdivided into the following phases:

• Reaching: the subject lifts the hand from the table to reach the object to be grasped.

• Manipulation: the subject grasps and lifts the object.

• Transport: the subject moves the object to the final position.

• Release: the subject releases the object.

• Return: the subject returns the hand to the starting position.

Gross movements tasks do not imply the interaction with any object, so items 17 to 19 were divided into two phases only: transport and return.

In order to implement an algorithm aimed at extracting the instants of onset and termination of each sub-movement from IMU signals, a preliminary comparison was performed between data extracted from the inertial sensor and data recorded by the optoelectronic system. Only data related to the 4 healthy subjects tested with both systems simultaneously were considered for this analysis.

**Figure 2:**
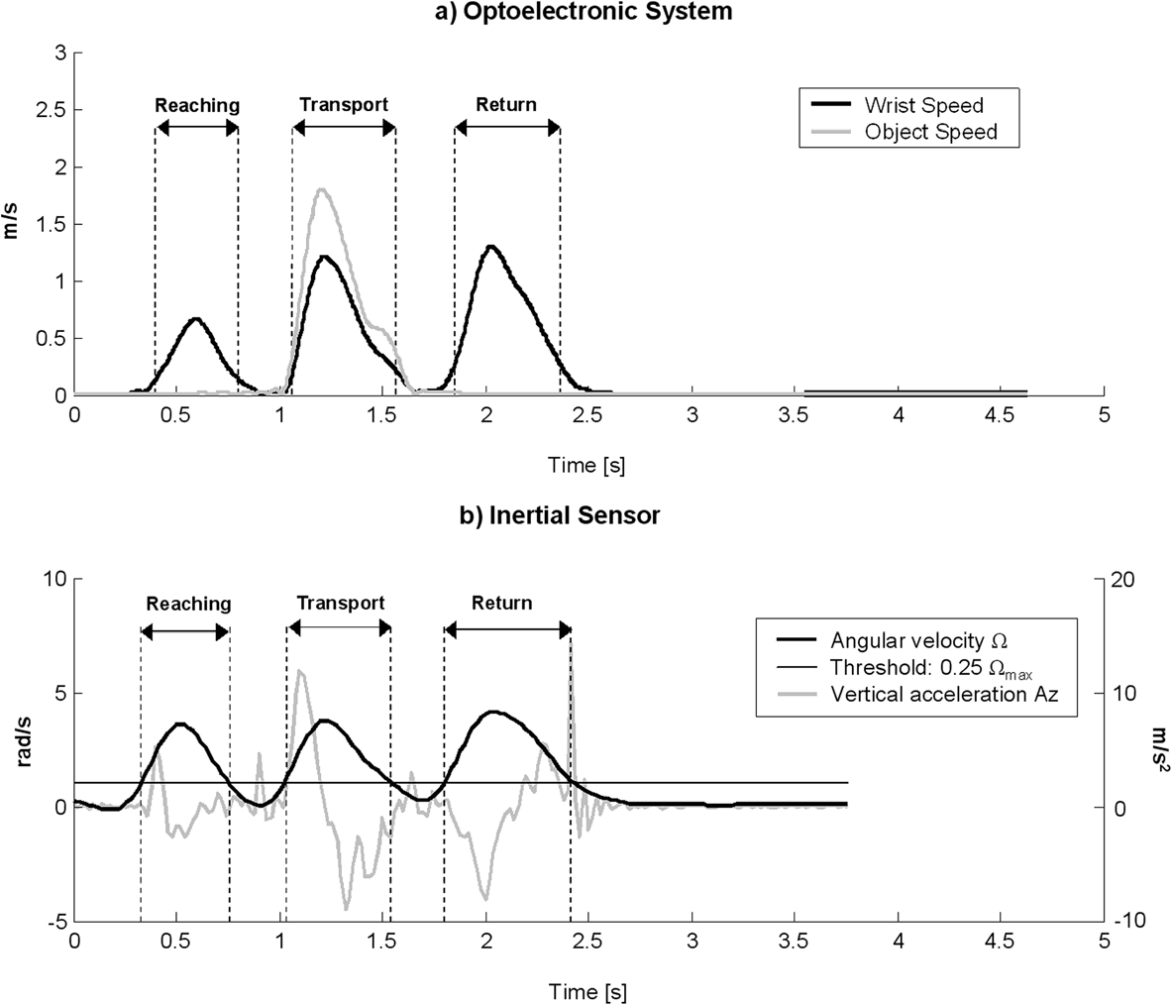
**Kinematic signals from the optoelectronic system and the inertial sensor.** Example of kinematic signals recorded from a healthy subject executing item 13 of ARAT. **(a)** Velocity profiles of markers positioned on the wrist and on the object. Data were extracted from the optoelectronic system. **(b)** Time courses of angular velocity and vertical linear acceleration extracted from the inertial sensor. Task sub-phases (reaching, transport and release) computed from the two systems are shown.

Markers coordinates recorded by Smart system were low-pass filtered (5^th^ order zero-lag Butterworth filter, cut-off frequency: 6 Hz) and differentiated to calculate wrist and object velocity. As shown in Figure 2a, the velocity profile of wrist marker (black line) is made up of three consecutive bell-shaped portions representing the three main arm sub-movements: reaching, transport and return. Transport phase is distinguishable also by looking at the velocity profile of the marker placed on the object (gray line). Instants of onset and termination of the transport phase were calculated as the first instants in which object speed exceeded and fell below a threshold of 20% of peak speed, respectively. Initiation and termination of reaching and return phases were similarly computed, considering the first and the last bell-shaped portions of wrist velocity profile, respectively. The threshold value was chosen to identify primary movements and exclude eventual initial adjustments of the arm [38]. As for manipulation phase, instants of onset and termination were in correspondence of the end of reaching and the beginning of transport, respectively. Similarly, onset and termination of release phase were sat equal to the end of transport and the beginning of return phase, respectively.

After optoelectronic data segmentation, IMU signals from accelerometers and gyroscopes were visually inspected and compared with the kinematic data related to wrist and object markers. The selected signals for data segmentation were, therefore, the vertical acceleration and the norm of angular velocity (Ω), the latter showing a triphasic profile very similar to the time-course of wrist marker velocity (see Figure 2b). Before Ω computation, signals from gyros were smoothed with a 4th order, zero-lag, low-pass Butterworth filter with a cut-off frequency of 2.5 Hz. In this preliminary study, a semi-automated procedure was proposed to extract instants of onset and termination of reaching, transport and return phases from the selected signals. In particular, the algorithm consisted in an initial manual phase, in which the operator visually inspected the Ω profile and roughly subdivided the signal into three distinct portions including the considered sub-movements. This operation was performed by using *ginput* function of Matlab. After this preliminary rough segmentation, the algorithm separately processed the three sub-signals and automatically calculated the temporal instants of interest. As shown in Figure 2b, the instant of termination of the return phase, that coincides with the first contact of the hand with the table surface, was calculated as the time frame corresponding to the last peak of the vertical acceleration. All the other instants (onset of reaching, transport and return, and termination of reaching and transport) were calculated by applying a suitable threshold to the angular velocity. In particular, four thresholds were considered, corresponding to increasing percentages of maximum angular velocity (Ω_max_): 0.15Ω_max_, 0.20Ω_max_, 0.25Ω_max_, 0.30Ω_max_. All four thresholds were applied to the three main portions of Ω profile separately and, then, instants of onset and termination were calculated as the first instants in which angular velocity exceeded and fell below the specific threshold, respectively. Absolute errors between temporal frames extracted from the optoelectronic data and the corresponding instants computed from gyros’ signals were calculated for the 4 thresholds, separately. Results are reported in Table 3. Mean data related to all instants pooled together showed that the threshold corresponding to 0.25 Ω_max_. induced the smallest absolute error between the two systems (0.046 s ± 0.037 s). This threshold was therefore selected and used to segment data of all subjects. Besides, mean absolute errors related to the termination of the return phase (calculated from vertical acceleration) were 0.041 s ± 0.034 s.

**Table 3:** Absolute errors between the inertial sensor and the optoelectronic system

		**TH 0.15**	**TH 0.20**	**TH 0.25**	**TH 0.30**
Absolute Error [s]	T0 reaching	0.083 (0.033)	0.064 (0.035)	0.050 (0.031)	0.038 (0.028)
	T1 reaching	0.060 (0.032)	0.068 (0.046)	0.054 (0.031)	0.113 (0.061)
	T0 transport	0.048 (0.029)	0.039 (0.026)	0.046 (0.039)	0.050 (0.045)
	T1 transport	0.079 (0.076)	0.081 (0.072)	0.050 (0.048)	0.155 (0.150)
	T0 return	0.089 (0.071)	0.064 (0.072)	0.029 (0.032)	0.047 (0.059)
	**All instants**	**0.072 (0.054)**	**0.063 (0.055)**	**0.046 (0.037)**	**0.080 (0.092)**

Values are mean (standard deviation). TH *n* (*n* = 0.15, 0.20, 0.25, 0.30) represent the 4 angular velocity thresholds used for task segmentation. T0: movement onset; T1: movement termination.

### Extraction of quantitative parameters

After segmentation of IMU signals, the following parameters were calculated for each task and for each sub-movement.

(1) $$Z\_P_{\mathrm{ijk}}=\frac{P_{i,j,k}-\bar{P_{\mathrm{co},j,k}}}{\sigma_{\mathrm{co},j,k}}$$

where *P* = parameter (duration, jerk), *i* = subject, *j* = ARAT task (item 1–19), *k* = task sub-movement (reaching, manipulation, transport, release, return), $\bar{P_{\mathrm{co},j,k}}$ = mean value of control group for parameter *P,* item *j* and sub-movement *k*, σ_*co,j,k*_ = standard deviation of control group for parameter *P*, item *j* and sub-movement *k*. Z-scores were calculated to quantify the deviation of each parameter from normative data.

• Duration: time elapsed between movement onset and termination.

• Jerk Index: logarithm of the mean amplitude of jerk (norm of the first time derivative of the acceleration), normalized with respect to the mean absolute acceleration and the duration of movement [6,8,39]. Before jerk computation, accelerometric signals were transformed to an absolute horizontal-vertical coordinate system by means of the rotation matrix provided by the IMU. This operation allowed the subtraction of the gravity component. Jerk Index was calculated to evaluate movements’ smoothness.

• Z-score: for all ARAT task and sub-movements, z-score related to duration (Z_Duration) and jerk index (Z_Jerk) were computed following Equation 1:

### Data analysis

To evaluate the existence of sub-groups of MS subjects with different levels of upper limb impairment, a hierarchical agglomerative cluster analysis was performed on clinical scores (ARAT, 9HPT and FTRS) after their standardization (i.e. subtraction of the mean and division by the standard deviation). The cluster analysis was conducted using the Euclidean distance measure and the average linkage clustering method (UPGMA).

Quantitative parameters extracted from IMU signals were described by median/range values and analyzed by means of statistical non-parametric methods. In particular, differences between MS subjects and healthy controls were analyzed with Mann–Whitney U Test (MWt), while comparisons among sub-groups of MS subjects with different levels of upper limb impairment were performed using Kruskal-Wallis test (KWt) and Bonferroni-Holm post-hoc procedure in presence of significant differences (p < 0.05). Finally, differences among the four ARAT sections were evaluated by Friedman test (Ft). The same post-hoc test was performed in case of significant differences.

The validity of the proposed method for evaluating upper limb impairment was investigating through a correlation analysis between the computed z-scores and the clinical scales. Spearman correlation coefficient *r* and the related p-value were therefore calculated.

## Results

### Clinical parameters

**Figure 3:**
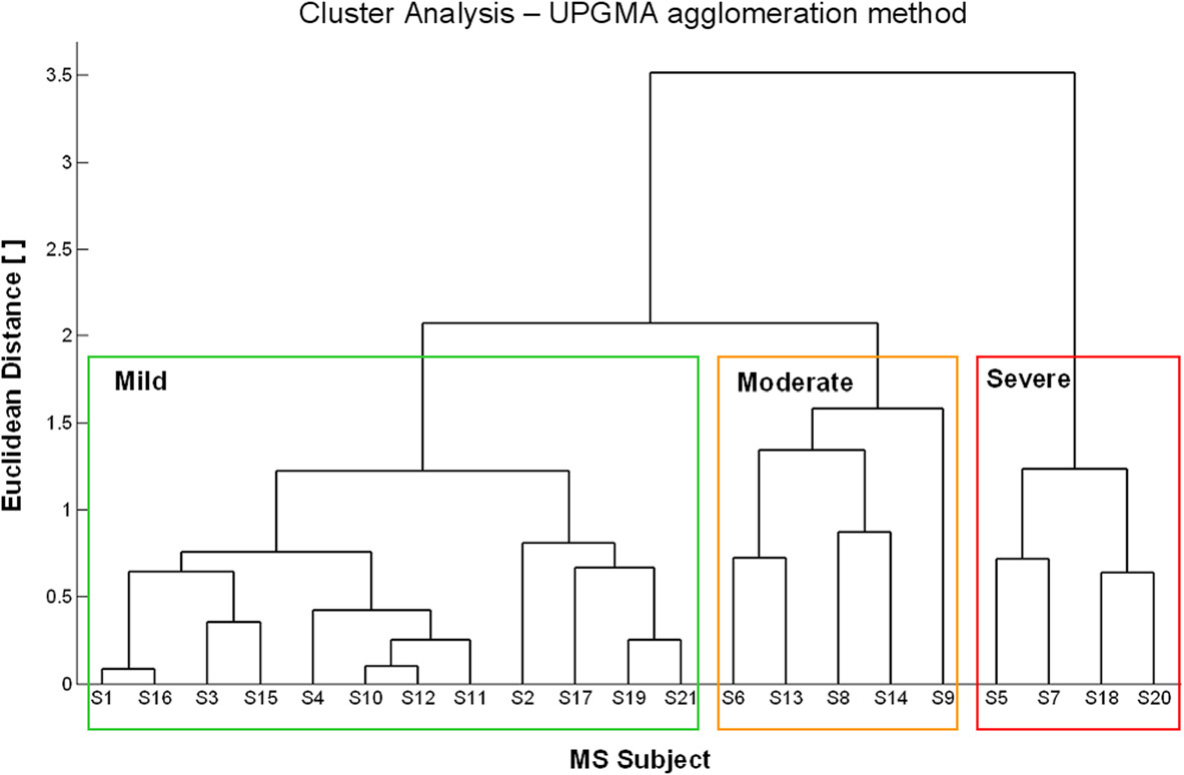
**Results of the cluster analysis.** Dendrogram of empirical sub-groups of MS subjects derived from hierarchical cluster analysis on clinical scores. Three clusters of subjects characterized by different level of upper limb dysfunction (mild, moderate and severe) were identified.

Clinical scores obtained by MS subjects and results of the cluster analysis are reported in Table 4 and Figure 3, respectively. All patients presented with reduced hand dexterity, as shown by 9HPT scores that were always higher than the threshold value typical of healthy adults with comparable age (19 s ± 2 s [40]). Two subjects (S5 and S7) were not able to perform 9HPT; for this reason the number of pegs moved per minute was also computed, so that a score equal to 0 was assigned to these patients. Results related to the cluster analysis performed on ARAT, 9HPT and FTRS ratings (see Figure 3) revealed the existence of three sub-groups of MS subjects with different level of upper limb impairment: mild (n = 12), moderate (n = 5) and severe (n = 4).

**Table 4:** Clinical assessment of MS subjects

**Group**	**Subject**	**ARAT [points]**	**9HPT [s]**	**9HPT [peg/min]**	**FTRS Post. + Int. [points]**	**FTRS Postural [points]**	**FTRS Intention [points]**
Mild impairment	S1	57	37.5	14.4	1	0	1
	S2	56	25.0	21.6	2	1	1
	S3	57	27.3	19.8	1	1	0
	S4	57	28.2	19.2	0	0	0
	S10	55	32.4	16.8	0	0	0
	S11	57	32.8	16.2	0	0	0
	S12	55	31.2	17.4	0	0	0
	S15	57	30.7	17.4	1	1	0
	S16	57	36.2	15.0	1	1	0
	S17	54	31.0	17.4	3	1	2
	S19	55	33.1	16.2	2	1	1
	S21	56	30.5	18.0	2	1	1
	**Median**	**56.5**	**31.1**	**17.4**	**1**	**1**	**0**
	**Non-outlier range**	**54-57**	**25.0-37.5**	**14.4-21.6**	**0-3**	**0-1**	**0-2**
Moderate impairment	S6	51	49.9	10.8	3	2	1
	S8	39	40.9	13.2	2	1	1
	S9	40	44.9	12.0	4	2	2
	S13	49	59.6	9.0	2	1	1
	S14	44	44.9	12.0	1	0	1
	**Median**	**44**	**44.9**	**12.0**	**2**	**1**	**1**
	**Non-outlier range**	**39-51**	**40.9-59.6**	**9.0-13.2**	**1-4**	**0-2**	**1-2**
Severe impairment	S5	34	Not able	0.0	4	1	3
	S7	37	Not able	0.0	3	1	2
	S18	34	105.9	5.4	5	2	3
	S20	39	159.5	3.6	5	1	4
	**Median**	**35.5**	**132.7**	**1.8**	**4.5**	**1**	**3**
	**Non-outlier range**	**34-39**	**105.9-159.5**	**0.0-5.4**	**3-5**	**1-2**	**2-4**
Whole sample	**Median**	**55**	**36.2**	**15.0**	**2**	**1**	**1**
	**Non-outlier range**	**34-57**	**25.0-59.6**	**3.6-21.6**	**0-5**	**0-2**	**0-4**

Subjects are grouped on the basis of the level of arm impairment (first column) following the results of the cluster analysis (Figure 3).

ARAT: Action Research Arm Test; 9HPT: Nine Hole Peg Test; FTRS: Fahn Tremor Rating Scale.

Mild subjects showed slight upper limb dysfunction mainly related to hand dexterity, as demonstrated by 9HPT (between 25 and 38 seconds) and ARAT which showed no dysfunction in six subjects (score: 57) and mild alteration of Pinch/Grip items in the remaining six patients (score: 54–56). Tremor was absent in four subjects, while the other eight patients showed mild/moderate postural end/or intention tremor (score: 1–2).

Subjects with moderate upper limb dysfunction showed more marked reduction of hand dexterity (9HPT: 41–60 s) and significant upper limb disorders (ARAT: 39–51) involving not only Grip and Pinch items but also Grasp and Gross tasks. A mild/moderate tremor was present in all five subjects.

Severe patients presented with a consistent reduction of both distal and proximal upper limb function, showing alterations in almost all 19 ARAT items and 9HPT scores above 100 s. Tremor was present in all subjects, especially for the intentional component that was severe (score: 3–4) in three subjects.

### Instrumental parameters

#### MS subjects *versus* healthy controls

Preliminary comparison between healthy subjects performing ARAT with their dominant side (n = 6) and those using the non-dominant arm (n = 6) did not reveal significant differences either in task duration (p_MWt_ = 0.936) and jerk index (p_MWt_ = 0.199). For this reason data describing the performance of control subjects were pooled together.

**Figure 4:**
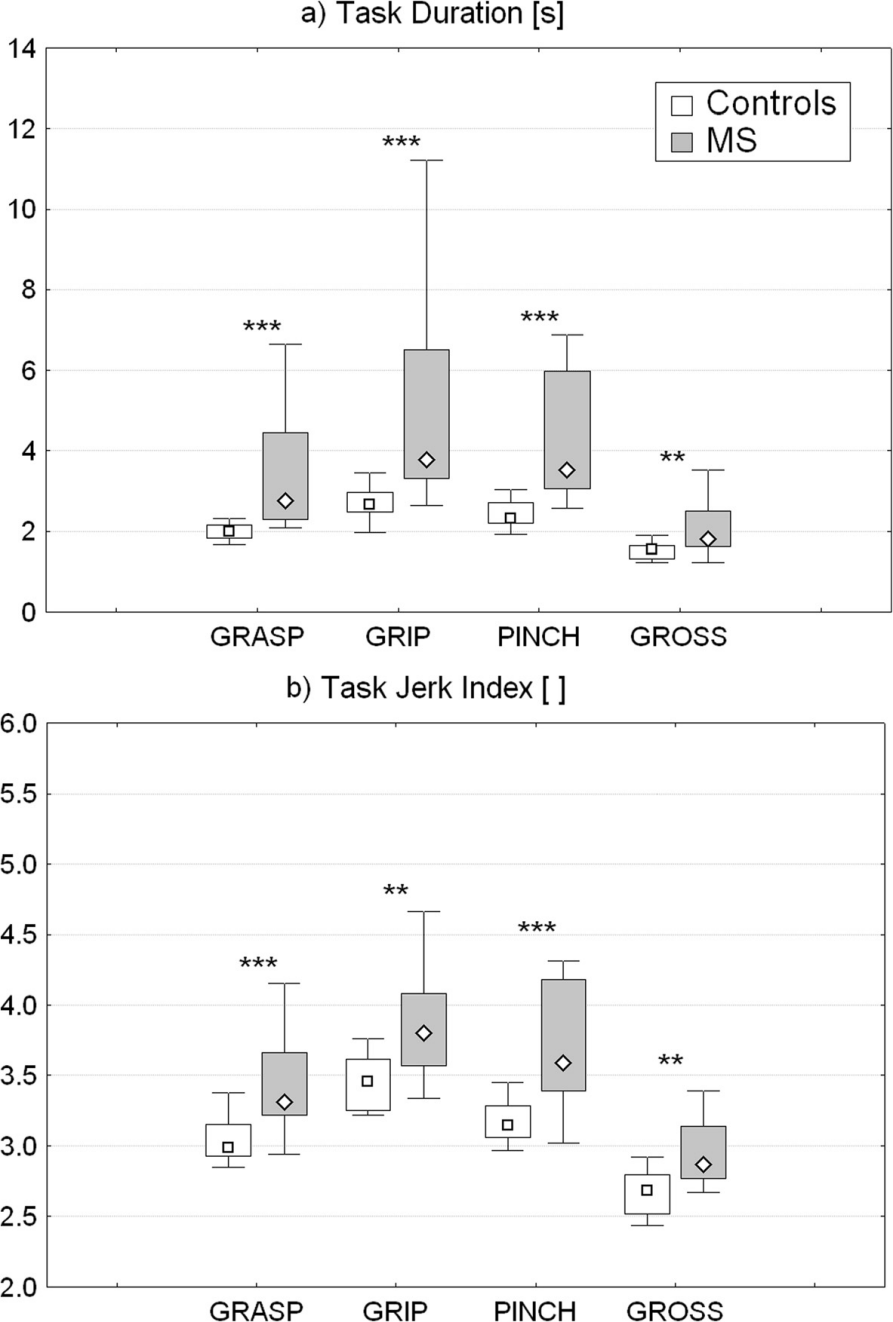
**Task duration and jerk index.** Instrumental parameters related to the mean task duration **(a)** and jerk index **(b)** of the four ARAT sections (Grasp, Grip, Pinch and Gross movement) for control and MS groups. Point: median. Box: interquartile range. Whisker: non-outlier range. **p < 0.01, ***p < 0.001 (Controls *versus* MS, Mann–Whitney U Test).

Quantitative parameters extracted from the inertial sensor during ARAT execution revealed that the movements executed by MS patients in all 4 test sub-sections were significantly slower (Figure 4a) and more jerky (Figure 4b) with respect to controls. As reported in Table 5, movement velocity and smoothness showed a mild but statistically significant decrease in all test sections also in the subgroup of six MS subjects who obtained maximum ARAT score (i.e. normal upper limb function).

**Table 5:** Comparison between healthy controls and MS subjects with subtle arm impairment – ARAT sections

		**Controls**	**MS**_**maxARAT**_	**p(MWt)**
**GRASP**	Task Duration [s]	2.0 (1.7-2.3)	2.4 (2.1-2.5)	0.018
	Task Jerk Index [ ]	3.0 (2.9-3.4)	3.2 (3.0-3.3)	0.042
**GRIP**	Task Duration [s]	2.7 (2.0-3.5)	3.3 (2.6-4.1)	0.049
	Task Jerk Index [ ]	3.5 (3.2-3.8)	3.7 (3.4-4.0)	0.034
**PINCH**	Task Duration [s]	2.3 (1.9-3.0)	2.8 (2.6-3.6)	0.035
	Task Jerk Index [ ]	3.1 (2.9-3.4)	3.3 (3.1-3.7)	0.029
**GROSS**	Task Duration [s]	1.6 (1.2-1.9)	1.7 (1.5-2.1)	0.035
	Task Jerk Index [ ]	2.7 (2.4-2.9)	2.8 (2.8-3.4)	0.024

Values are median (non-outlier range).

MS_maxARAT_: MS subjects evaluated with maximum ARAT score (i.e. normal upper limb function); MWt: Mann–Whitney U Test (Controls vs MS_maxARAT_).

**Figure 5:**
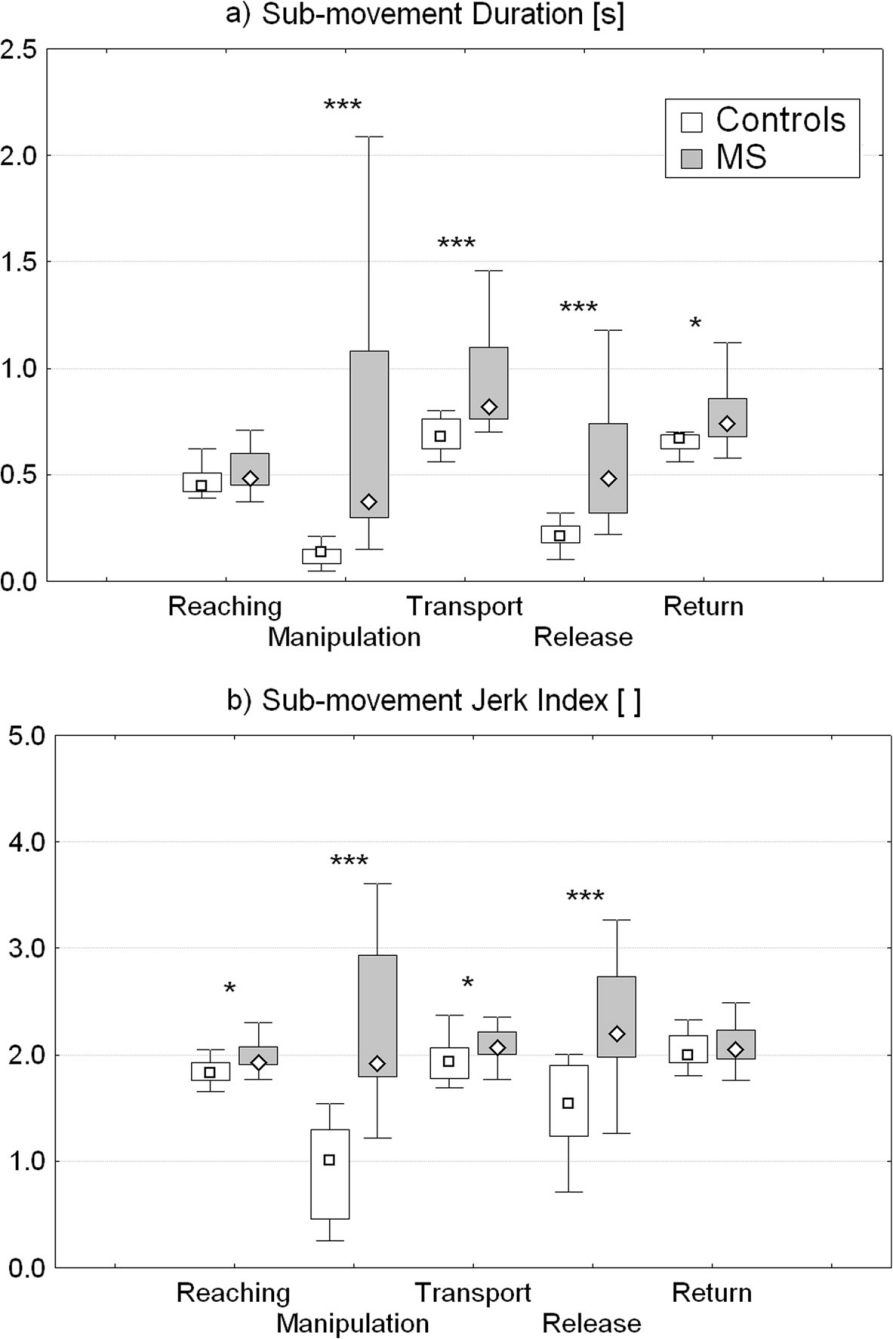
**Sub-movement duration and jerk index.** Instrumental parameters related to the duration **(a)** and jerk index **(b)** of the basic sub-movements involved in ARAT tasks (reaching, manipulation, transport, release and return) for control and MS groups. Point: median. Box: interquartile range. Whisker: non-outlier range. *p < 0.05, ***p < 0.001 (Controls *versus* MS, Mann–Whitney U Test).

**Figure 6:**
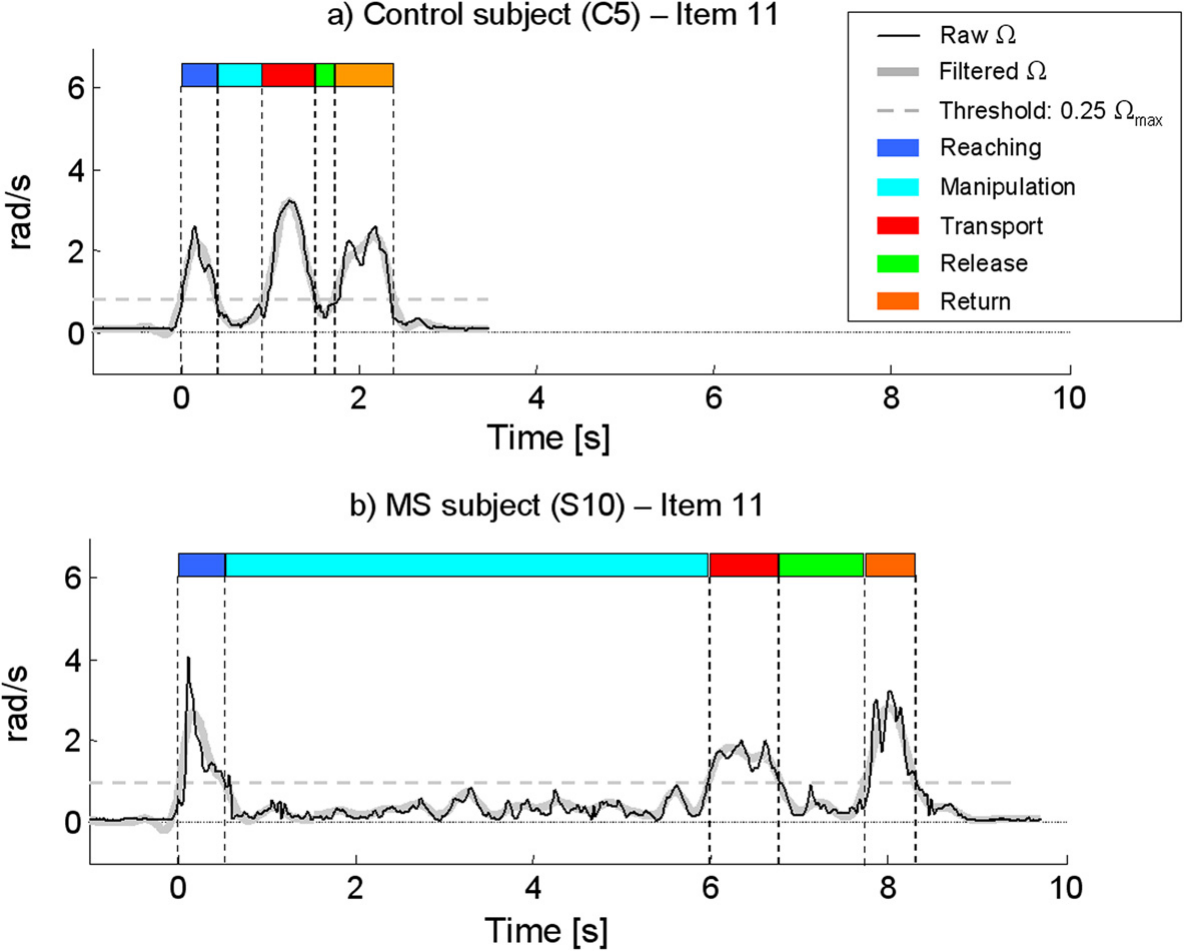
**Examples from two subjects.** Raw and filtered angular velocity profiles of a representative healthy control **(a)** and a subject with MS **(b)** executing item 11 of ARAT. Task sub-movements related to the two subjects are shown.

A more detailed analysis of task sub-movements executed by the whole group of MS patients showed that the above alterations were mainly related to manipulation, transport and release phases which were characterized by significantly higher duration (Figure 5a) and jerk index (Figure 5b) with respect to healthy subjects. The most affected sub-movement was manipulation, which showed a median percentage increase with respect to control data of 174% for duration and 90% for jerk index. Contrarily, reaching and return phases revealed mild differences between groups. As reported in Table 6, a mild but statistically significant prolongation of manipulation, transport and release phases was noticed also in the six MS subjects having maximum ARAT score. Again, manipulation phase was the most impaired sub-movement, showing not only an increase of duration but also a mild but significant reduction of smoothness. Figure 6 shows the angular velocity profiles of one healthy control (Figure 6a) and one MS subject (Figure 6b) recorded during the execution of an ARAT task (item 11). It can be noticed how the movement of the MS patient is more jerky and prolonged especially in the manipulation phase.

**Table 6:** Comparison between healthy controls and MS subjects with subtle arm impairment – ARAT task sub-movements

		**Controls**	**MS**_**maxARAT**_	**p(MWt)**
**Reaching**	Phase Duration [s]	0.45 (0.39-0.62)	0.46 (0.41-0.53)	0.814
	Phase Jerk Index [ ]	1.83 (1.66-2.05)	1.83 (1.63-1.96)	0.742
**Manipulation**	Phase Duration [s]	0.14 (0.05-0.21)	0.30 (0.28-0.37)	0.002
	Phase Jerk Index [ ]	1.01 (0.25-1.54)	1.77 (1.22-1.92)	0.003
**Transport**	Phase Duration [s]	0.66 (0.56-0.80)	0.77 (0.70-0.90)	0.020
	Phase Jerk Index [ ]	1.93 (1.69-2.37)	1.96 (1.77-2.02)	0.687
**Release**	Phase Duration [s]	0.21 (0.10-0.32)	0.28 (0.22-0.32)	0.028
	Phase Jerk Index [ ]	1.52 (0.71-2.01)	1.77 (1.26-2.20)	0.209
**Return**	Phase Duration [s]	0.67 (0.56-0.70)	0.68 (0.62-0.82)	0.476
	Phase Jerk Index [ ]	2.00 (1.80-2.33)	1.97 (7.76-2.23)	0.541

Values are median (non-outlier range).

MS_maxARAT_: MS subjects evaluated with maximum ARAT score (i.e. normal upper limb function); MWt: Mann–Whitney U Test (Controls vs MS_maxARAT_).

**Figure 7:**
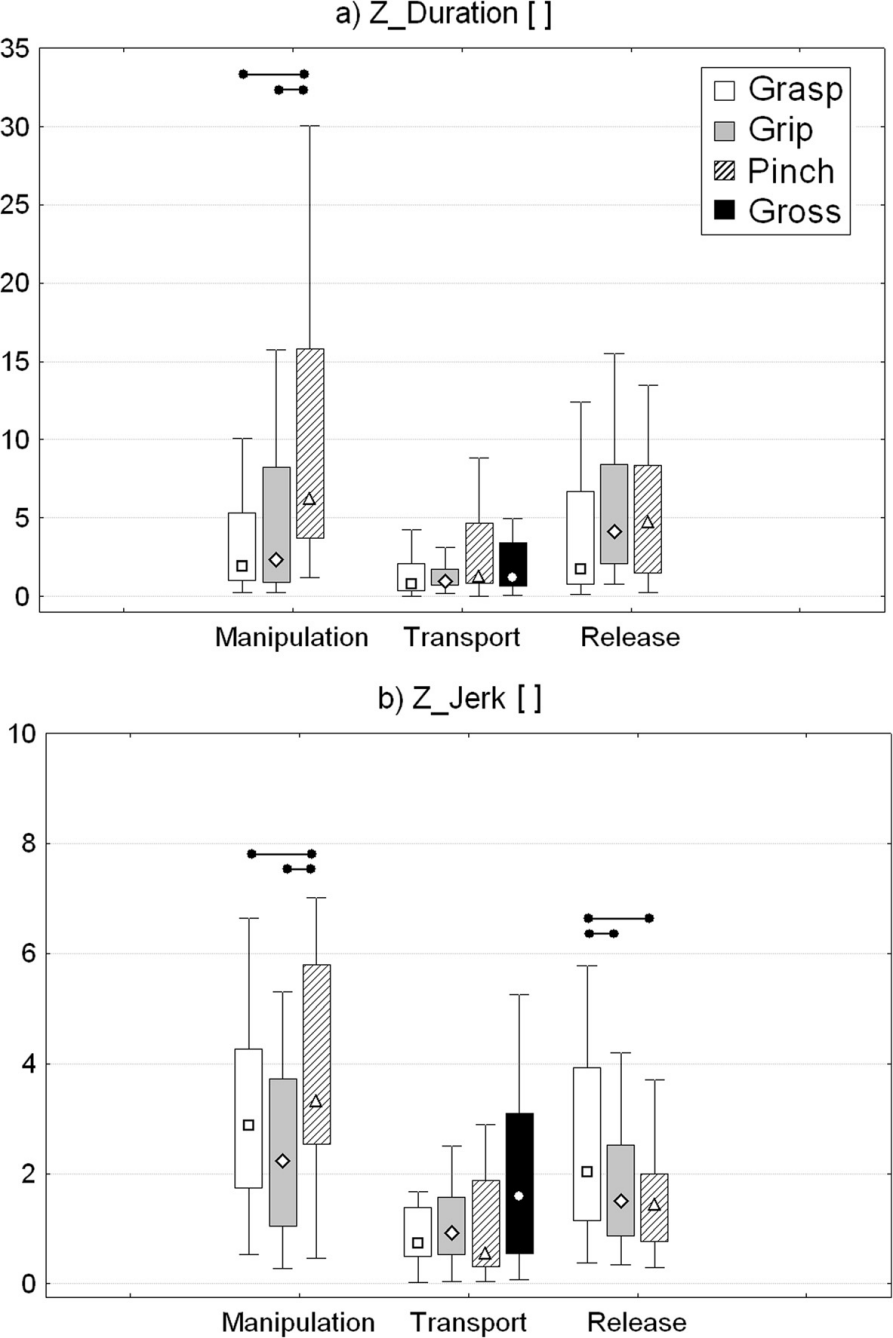
**Comparison among ARAT sections.** Z-scores related to the duration **(a)** and the jerk index **(b)** of manipulation, transport and release phases for Grasp, Grip, Pinch and Gross movement sections. Point: median. Box: interquartile range. Whisker: non-outlier range. Horizontal lines and dots indicate significant differences between two phases (p < 0.05, Bonferroni-Holm post-hoc comparison).

Results of the comparison among ARAT sections in the three most affected sub-movements (manipulation, transport and release) are reported in Figure 7 in terms of z-scores (Z_Duration and Z_Jerk), that represent the deviation of the specific parameter from normative data. Significant differences among ARAT tasks were found in the manipulation phase for both Z_Duration (p_Ft_ < 0.001) and Z_Jerk (p_Ft_ = 0.005). Post hoc analysis revealed that Pinch items were characterized by the greatest deviations from control values. Transport phase was similar in all ARAT sections, while a significant difference was found in release phase (p_Ft_ < 0.001), with Grasp items showing the greatest jerk index.

### Validity

**Figure 8:**
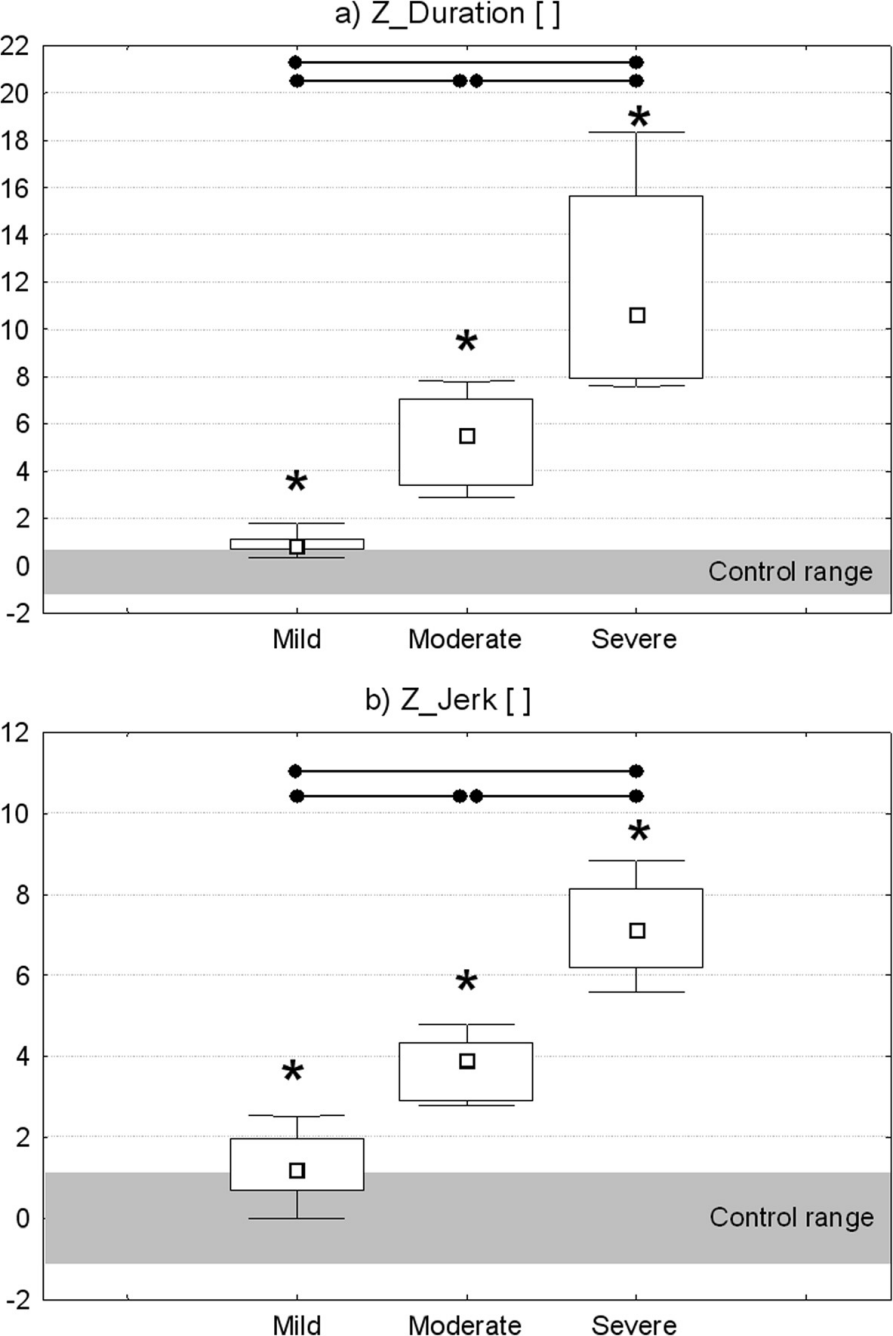
**Comparison among different levels of upper limb impairment.** Z-scores related to the mean task duration **(a)** and jerk index **(b)** for the three subgroups of MS subjects showing increasing levels of upper limb impairment (Mild, Moderate and Severe). Point: median. Box: interquartile range. Whisker: non-outlier range. Horizontal lines and dots indicate significant differences between two groups (p < 0.05, Bonferroni-Holm post-hoc comparison). Asterisks indicate significant differences with respect to healthy control group (p < 0.05, Mann–Whitney U Test).

A significant negative correlation was found between ARAT score and z-scores related to the mean item duration (r = -0.823, p < 0.001) and jerk index (r = -0.898, p < 0.001). The two instrumental parameters were also negatively correlated with 9HPT score expressed as number of pegs per minute (Z_Duration: r = -0.776, p < 0.001; Z_Jerk: r = -0.765, p < 0.001). As shown in Figure 8, a statistically significant difference among the three sub-groups of MS subjects with increasing levels of upper limb dysfunction (mild, moderate and severe) was found in both Z_Duration (p_KWt_ < 0.001) and Z_Jerk (p_KWt_ < 0.001). Post hoc test demonstrated that each group was significantly different from the others in both parameters. In particular, mild subjects showed the lowest deviation from normative data (anyway significantly higher than control values), followed by moderate and severe patients.

**Figure 9:**
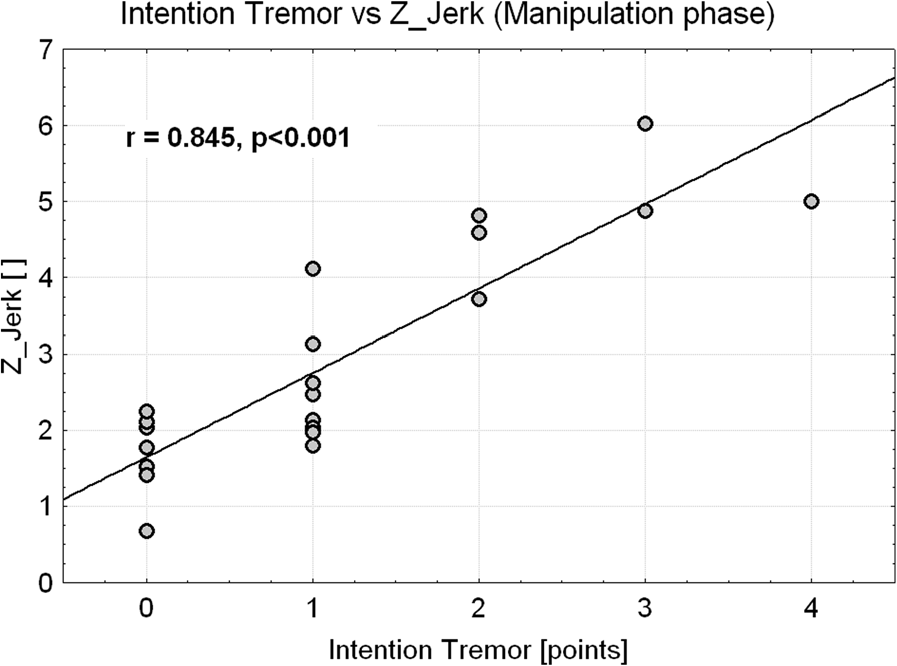
**Intention tremor *versus *Z_Jerk.** Scatter plot showing the correlation between the level of intention tremor rated with FTRS and the Z_score related to the manipulation phase jerk. Points represent MS subjects. Spearman correlation coefficient *r* is shown.

As shown in Figure 9, the level of intention tremor evaluated with FTRS was positively correlated with Z_Jerk calculated on manipulation phase (r = 0.845, p < 0.001). Interestingly, the group of seven MS subjects who received a score equal to 0 (i.e. absence of intention tremor) showed a significantly higher Z_Jerk with respect to healthy controls [Control median (range): 0.2 (-1.5 - 1.4); MS_NoTremor_ : 1.8 (0.7 - 2.2); p_MWt_ = 0.001]. Intention tremor was also correlated with Z_Jerk calculated on release phase (r = 0.801, p < 0.001) and, to a lesser extent, with Z_Jerk related to transport phase (r = 0.593, p = 0.005). Analysis of these two instrumental parameters did not reveal any statistically significant difference between healthy subjects and MS_NoTremor_ group.

## Discussion

In the present study, upper limb function of healthy subjects and persons with MS was analyzed via a single inertial sensor on the wrist, during the execution of ARAT. This test was chosen among the others clinical scales because it is one of the most widely used standardized measures for upper limb, it is relatively quick and, at the same time, it evaluates both arm and hand during the execution of functional tasks very similar to the activities of daily living. Nevertheless, ARAT suffers of three major drawbacks: 1) its outcome is limited to a subjective score describing the quality of the performance, 2) a ceiling effect is present, making impossible the detection of possible improvements induced by rehabilitation treatments in mild subjects scored at the top of the scale, 3) the score does not provide indications about disorders in the task sub-movements. An instrumented ARAT aims to partly overcome these limitations by making use of a measurement system to compute quantitative parameters to more subtly investigate upper limb functionality in persons with MS.

In the present study, instrumented ARAT was applied on MS subjects by instructed physiotherapists in a typical physical rehabilitation gym. Test administration required approximately 15 minutes: 5 minutes for preparation and 10 minutes for the test execution. MS patients did not report any discomfort in executing the instrumented test and physical therapists indicated that the setting-up procedure was quick and easy to be performed, suggesting that the method is applicable in a clinical setting. The proposed algorithm for task segmentation was able to detect the instants of onset and termination of each phase with an average absolute error of 0.046 s with respect to the optoelectronic system assumed as a gold standard. This error was considered acceptable for the aim of this work, but further studies are required to extend the accuracy analysis also to MS subjects.

The results of the study showed that this method is able to discriminate motor performances of persons with MS from that of healthy subjects. In particular, movements executed by MS patients were significantly prolonged and less smooth with respect to controls in all ARAT items, thus revealing significant alterations in the execution of three-dimensional functional tasks involving both arm and hand. These results, in turn, enforced those already found in previous studies analyzing less complex bi-dimensional reaching movement of proximal arm by means of a graphic tablet [41] and a planar robotic manipulandum [5,6,8,38]. With respect to the traditional ARAT, a first novel supplementary information provided by the instrumented test is the quantification of movement smoothness by means of jerk index, that is widely regarded as a measure of the ability to control coordinated multi-joint movements [39]. In particular, the significant decrease of smoothness characterizing the movements of MS subjects may be partly due to tremor, as a strong positive correlation was found between Z_Jerk and tremor level measured with FTRS. Moreover, increased jerk could be ascribed to the frequent corrections of movement direction that might be related to a dysfunction in sensory input integration [42] and/or to the attempt to compensate for poor proprioceptive control of upper limb with longer-loop visual feedback [43,44].

A second relevant information provided by instrumented test is the quantitative characterization of the basic sub-movements involved in each ARAT task (i.e. reaching, manipulation, transport, release and return). These further data may represent an added value since clinical tests do not provide this information that is essential to understand upper limb physiology and physiopathology [45]. Even though finger movements are not directly measured by the wrist-mounted sensor, the separate analysis of task sub-phases allows to obtain, within a single test, not only a quantitative assessment of proximal arm function but also an indirect measure of manual dexterity, thus providing additional information useful to obtain more detailed indications about patient’s specific deficit and, consequently, to select the most suitable rehabilitation treatment aimed at improving arm transportation and/or hand function. In particular, analysis of the sub-movements revealed that upper limb impairment in MS subjects was mainly related to manipulation, transport and release phases. The greatest alterations with respect to healthy subjects were found in manipulation phase which was significantly impaired in all ARAT tasks, suggesting a consistent deficit in interacting with both small and larger object. However, comparison between different tasks revealed that precision grip required by Pinch items was significantly more compromised with respect to gross manipulation involved in Grasp and Grip sections. Similar results were found in a previous study [8] and could be ascribed to different factors. First of all, the coordination of fine finger movements, that is a fundamental prerequisite for a stable precision grip [46,47] has been demonstrated to be impaired in MS subjects, even in the early stages of the disease [48]. Secondly, sensory deficits, mainly related to altered and/or reduced tactile sensibility are very common in subjects with MS [49,50] and may play a primary role in the reduction of feedback control of fingertip actions [51], especially during manipulation of small and light objects.

Importantly, the results of the present study showed that instrumented ARAT was able to detect subtle upper limb alterations not visible from the clinical score. In particular, analysis of the quantitative parameters extracted by the inertial sensor revealed a mild but statistically significant increase of movement duration and jerk also in MS subjects who obtained maximum ARAT score, that means normal upper limb function. These alterations were present in all ARAT sections thus suggesting that mild MS subjects presented not only with reduced fine manual dexterity, as detected also by 9HPT, but also with subtle proximal arm anomalies emerged from Gross section analysis. This findings can be useful for two main reasons. Firstly, the high sensitivity of the proposed parameters in detecting subclinical upper limb alterations not revealed by traditional scales could be helpful to choose a suitable preventive intervention to slow down the progression of these symptoms even in the early stages of the disease. Secondly, the use of quantitative continuous parameters avoids the ceiling effect related to clinical ARAT score, thus allowing the detection of possible upper limb improvements also in mild subjects scored at top of the scale.

The validity of the method for evaluating upper limb impairment in MS subjects was studied by analyzing the correlations between clinical scales and z-scores representing the alteration of the instrumental parameters with respect to normative values extracted from the healthy control group. These scores related to mean task duration and jerk index were strongly correlated with both ARAT and 9HPT ratings. Moreover, the two parameters were able to discriminate between different levels of upper limb impairment, as demonstrated by the significant progressive increase of Z_Duration and Z_Jerk with increasing arm dysfunction. Taken together, these results suggested that instrumented ARAT is a valid tool for quantifying upper limb dysfunction in persons with MS. Interestingly, Z_Jerk calculated on manipulation, transport and release sub-movements were significantly correlated with FTRS score rating intention tremor. In agreement with the clinical definition of intention tremor (i.e. increase of kinetic tremor at the end of a goal-directed movement) the correlations with FTRS score were stronger during manipulation and release phases with respect to transport movement. Similar results were found by Feys et al. [43]. Moreover, a significant increase of Z_Jerk related to manipulation was found in MS subjects who received a FTRS score equal to 0 (i.e. absence of intention tremor), suggesting that this parameter can be a more sensitive measure of intention tremor during the execution of functional tasks very similar to the activities of daily living.

There are some limitations that need to be addressed regarding the present study. A first limitation is the small number of subjects included in the study. Instrumented ARAT should be applied on a greater number of patients in order to confirm these preliminary results about MS and to extend upper limb analysis to other pathologies, such as stroke and Parkinson’s disease. Future studies are also needed to test the reliability of the proposed parameters and to define the minimum significant detectable change of this method. Longitudinal studies should also be performed to determine if the instrumental parameters extracted by the inertial sensor might be sensitive descriptors of clinical progression. A second limitation concerns the proposed procedure for data segmentation that is semi-automated. More sophisticated and completely automated algorithms should be implemented to further simplify the analysis and to explore more properties of the sensor-derived measures. A third limitation consists in the absence of a direct measure of finger movements which are indeed very essential in completion of ARAT tasks. Future inclusion of finger-mounted sensors [27,52] could provide a more detailed assessment of hand function, which can complement the indirect measure obtained with the proposed method.

## Conclusion

In summary, the results of the present study showed that the proposed method i) is applicable in clinical settings, ii) is able to discriminate motor performances of MS subjects from that of healthy controls, iii) is able to discriminate between different levels of upper limb functional limitation in different task sub-movements and iv) is able to detect mild alterations not visible from clinical scores. Validity of the proposed instrumental parameters for evaluating upper limb impairment was also demonstrated. Even though caution must be taken given the small sample size, these preliminary findings suggest that the use of a single inertial sensor during the execution of ARAT could be a quick and easy-to-use method for a sensitive and more detailed quantitative characterization of upper limb function in persons with MS. Future inclusion of finger-mounted sensors could provide further indication about hand function in MS.

## Competing interests

The authors declare that they have no competing interests.

## Authors’ contributions

The overall design of the experiment was agreed by all authors after extensive discussions. DC selected the subjects, conducted the clinical evaluations and participated in instrumental data acquisition. IC implemented the algorithms for data processing, participated in instrumental data acquisition and performed data analysis. IC and DC performed data interpretation. MF participated in data interpretation. IC wrote the manuscript. DC and MF reviewed the manuscript. All authors read and approved the final manuscript.
